# Platelet-derived calpain cleaves the endothelial protease-activated receptor 1 to induce vascular inflammation in diabetes

**DOI:** 10.1007/s00395-020-00833-9

**Published:** 2020-12-01

**Authors:** Anastasia Kyselova, Amro Elgheznawy, Ilka Wittig, Juliana Heidler, Alexander W. Mann, Wolfram Ruf, Ingrid Fleming, Voahanginirina Randriamboavonjy

**Affiliations:** 1 Institute for Vascular Signalling, Centre for Molecular Medicine, Goethe University, Theodor-Stern-Kai 7, 60596 Frankfurt am Main, Germany; 2 German Center for Cardiovascular Research (DZHK), Partner site Rhein-Main, Frankfurt am Main, Germany; 3 Functional Proteomics, SFB 815 Core Unit, Goethe University, Frankfurt am Main, Germany; 4 Endokrinologikum Frankfurt, 60596 Frankfurt am Main, Germany; 5 Center for Thrombosis and Hemostasis, University Medical Center, Mainz, Germany; 6 Department of Immunology and Microbiology, The Scripps Research Institute, La Jolla, CA USA

**Keywords:** Endothelial cells, Microparticles, Endothelial protein C receptor, ICAM-1, Calpain

## Abstract

Diabetes mellitus is a major risk factor for cardiovascular disease. Platelets from diabetic patients are hyperreactive and release microparticles that carry activated cysteine proteases or calpains. Whether platelet-derived calpains contribute to the development of vascular complications in diabetes is unknown. Here we report that platelet-derived calpain1 (CAPN1) cleaves the protease-activated receptor 1 (PAR-1) on the surface of endothelial cells, which then initiates a signaling cascade that includes the activation of the tumor necrosis factor (TNF)-α converting enzyme (TACE). The latter elicits the shedding of the endothelial protein C receptor and the generation of TNF-α, which in turn, induces intracellular adhesion molecule (ICAM)-1 expression to promote monocyte adhesion. All of the effects of CAPN1 were mimicked by platelet-derived microparticles from diabetic patients or from wild-type mice but not from CAPN1^−/−^ mice, and were not observed in PAR-1-deficient endothelial cells. Importantly, aortae from diabetic mice expressed less PAR-1 but more ICAM-1 than non-diabetic mice, effects that were prevented by treating diabetic mice with a calpain inhibitor as well as by the platelet specific deletion of CAPN1. Thus, platelet-derived CAPN1 contributes to the initiation of the sterile vascular inflammation associated with diabetes via the cleavage of PAR-1 and the release of TNF-α from the endothelial cell surface.

**Electronic supplementary material:**

The online version of this article (10.1007/s00395-020-00833-9) contains supplementary material, which is available to authorized users.

## Introduction

Diabetes mellitus is a major risk factor for the development of cardiovascular disease(s), and the morbidity and mortality associated with diabetes are frequently related to micro- and macro-vascular complications, characterized by accelerated atherothrombosis [2]. Several mechanisms contribute to such a diabetes-associated prothrombotic state, including endothelial dysfunction, hypercoagulability and platelet hyperactivation [6, 45]. Although it is well accepted that platelets are involved in the regulation of vascular homeostasis, exactly how they contribute to changes in the vascular wall is not fully understood. One mechanism by which platelets affect the vascular wall is through the release of factors stored in platelet granules. For example, following their release from α-granules chemokines; such as CXCL4 and CCL5, are deposited on the endothelial cell surface to initiate monocyte recruitment and diapedesis [22]. Dense granule contents also appear to play a critical role in thrombosis and vascular remodeling, as Hermansky–Pudlak syndrome 3-deficient mice; which demonstrate impaired platelet dense-granule secretion, are protected from thrombotic arterial occlusion and the development of neointimal hyperplasia [21]. In addition to the release of soluble factors, platelets can also affect vascular homeostasis through the release of platelet-derived microparticles (PMPs) that contain proteins and microRNAs that can be transferred to the vasculature [44].

One group of platelet proteins that have been linked with platelet hyperactivation in the context of diabetes are the Ca^2+^-dependent cysteine proteases or calpains [39]. The latter are involved in several steps of platelet activation and calpain activation affects integrin signaling, aggregation, spreading, and granule secretion [24]. Interestingly, calpains can also be secreted by platelets and have been detected in PMPs [12, 33, 37]. Indeed, calpain activity in plasma correlates well with PMP levels and is significantly higher in plasma from diabetic subjects than from healthy volunteers [37]. The current study was designed to determine the relevance of platelet-derived calpain 1 (CAPN1) in the vascular complications associated with diabetes by identifying new calpain target proteins on the surface of endothelial cells. Use was made of CAPN1^−/−^ mice and mice lacking CAPN1 specifically in platelets (CAPN1^ΔPF4^ mice) to assess the importance of extracellular CAPN1 on endothelial cell activation and vascular inflammation.

## Methods

The authors declare that all supporting data are available within the article and it is Electronic Supplementary Material.

### Human subjects

A total of 30 patients with type 2 diabetes mellitus attending the clinic for routine control visits were included in the study (15 women, 15 men; mean age: 42.16 ± 2.39 years; age range: 25–60 years, hemoglobin (Hb) A1c > 8% and fasting plasma glucose of 8 ± 0.75 mmol/L). The patients were either without treatment or treated with metformin. A total of 34 nondiabetic, age-matched subjects served as the control group (18 women, 16 men; mean age: 39.1 ± 2.06 years; age range: 23–60 years; HbA1c, 5 ± 0.7%; fasting plasma glucose, 5 ± 0.35 mmol/L). All individuals claimed not to have taken any medication known to interfere with platelet aggregation for at least 10 days before the experiment. The study protocol was approved by the ethics committee of the Goethe University Hospital (No. E 61/09 geschäfts Nr 86/09) and all of the participants gave written informed consent.

### Animals

C57BL/6 mice (6–8 weeks of age) were purchased from Charles River Laboratories (Sulzfeld, Germany), and C57BL/6 PAR1^−/−^ mice [4], were bred at the animal facilities in Mainz. C57BL/6 CAPN1 knockout mice (CAPN1^−/−^) were generated as described [37], and mice lacking CAPN1 specifically in platelets (referred to as CAPN1^ΔPF4^ mice) were generated by crossing C57BL/6 floxed CAPN1 mice with PF4-deleter mice (C57BL/6-Tg(Pf4-icre)Q3Rsko/J; The Jackson Laboratory, Bar Harbor, Maine, USA). Male and female mice were used throughout. Wild-type and CAPN1^ΔPF4^ littermates were randomly allocated to receive saline or streptozotocin (STZ, 50 mg/kg i.p.) to induce diabetes, blood glucose was controlled after 3 and 9 weeks and mice were monitored for total of 12 weeks. Animals with fasting plasma glucose more than 250 mg/dL were considered diabetic and were included in the study. Group sizes were determined by a priori power calculation. In some experiments, healthy and diabetic C57BL/6 mice were randomly allocated to receive either vehicle or the calpain inhibitor A-705253 (30 mg/kg) in the drinking water for 12 weeks. In a second diabetic animal model, 6-week-old wild-type and CAPN1^ΔPF4^ littermates were fed a high-fat diet (34% fat, 23.8% sugar and 265 mg/kg cholesterol, E15742-34, Sniff, Soest, Germany) for 12 weeks. Mice that achieved a fasting plasma glucose over 250 mg/dL on week 12 were considered diabetic. Animals continued to receive the high-fat diet over an additional 8–12 weeks. All animals were housed in conditions that conform to the Guide for the Care and Use of Laboratory Animals published by the US National Institutes of Health (NIH publication no. 85–23). Both the university animal care committee and the Federal Authorities for Animal Research, Regierungspräsidium Darmstadt (Hessen, Germany) approved the study (study numbers: F28/17_44 and FU-1204).

### Statistical analysis

Data are expressed as mean ± SEM, and statistical evaluation was performed using GraphPad Prism 7 software. For all data, D’Agostino-Pearson omnibus normality test was performed to confirm normal distribution. For comparisons between two groups, a paired or unpaired Student’s *t* test was performed where appropriate. For comparisons between three or more groups, one- or two-way ANOVA followed by Tukey’s or Sidak’s multiple comparisons post-test were performed. Values of *p* < 0.05 were considered statistically significant. *p* values are given in the figures. Throughout the manuscript, representative images were selected as those that show values close to the means of the results obtained from all analyzed samples.

Detailed methods can be found in the Electronic Supplementary Material.

## Results

### Link between diabetes and the CAPN1-dependent shedding of EPCR

**Fig. 1:**
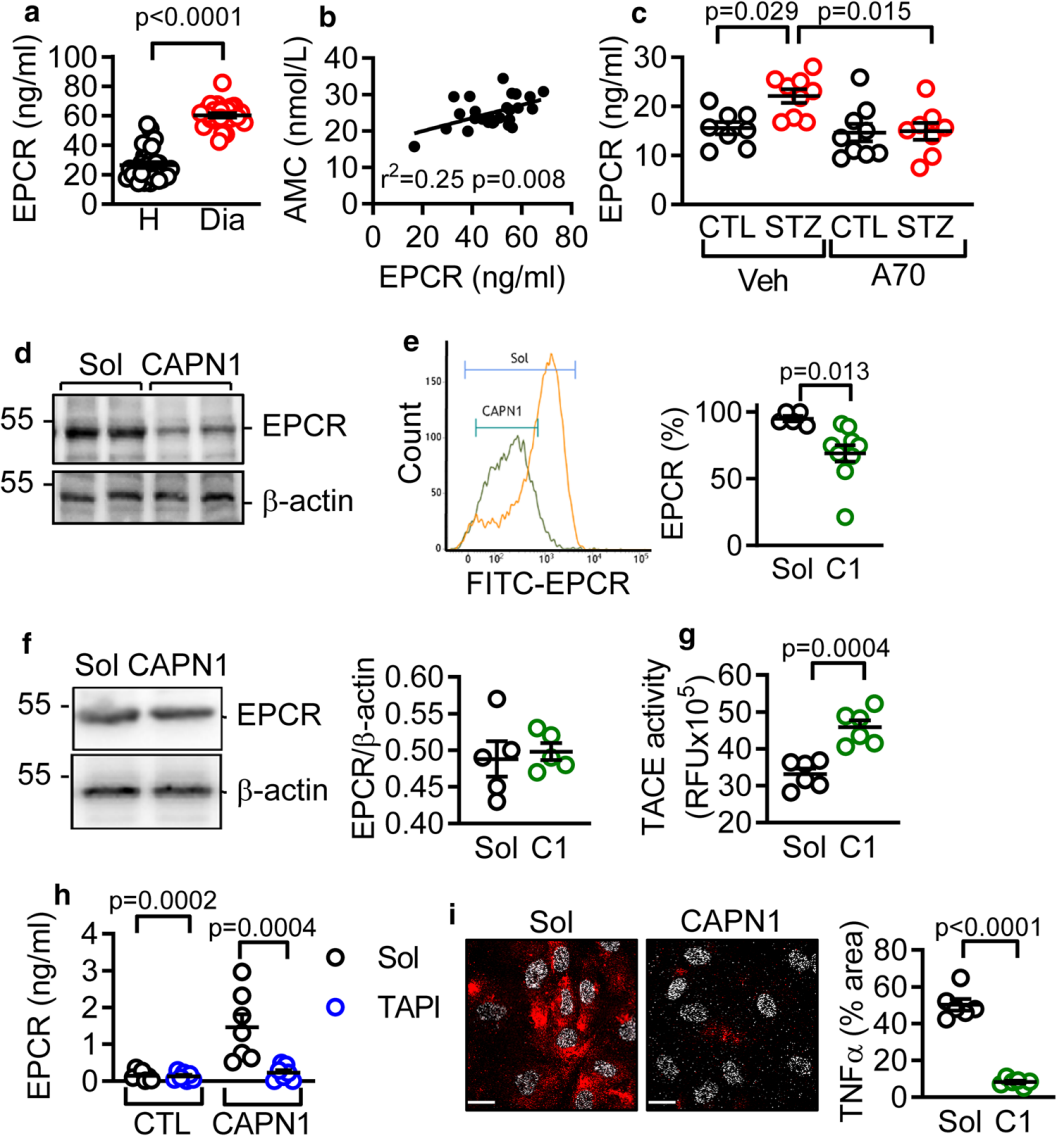
Diabetes-induced calpain activation enhances EPCR shedding. **a** Plasma levels of EPCR in samples from healthy (H, *n* = 34) and diabetic patients (Dia, *n* = 30); (Student’s *t* test). **b** Correlation between EPCR levels and calpain activity assessed as the generation of 7-amino-4-methylcoumarin (AMC) in human plasma; *n* = 26 individuals. **c** EPCR levels in plasma from healthy (CTL) and streptozocin (STZ) diabetic mice treated in vivo with vehicle (Veh) or A705232 (A70, 30 mg/kg/day) for 12 weeks; *n* = 9 animals per group (one-way ANOVA and Tukey’s post-test). **d** Representative blot showing EPCR levels in membrane preparations from human endothelial cells treated with either solvent or CAPN1 (C1; 0.3 U/ml, 4 h). The blot shown is representative of 4 additional experiments. **e** FACS analysis of EPCR levels on the surface of human endothelial cells treated with either solvent or CAPN1 (C1; 0.3 U/ml, 4 h); *n* = 5 different cell batches each studied in duplicate (Student’s *t* test). **f** EPCR in endothelial cell lysates treated with solvent (Sol) or CAPN1 (C1; 0.3 U/ml, 30 min); *n* = 5 independent cell batches. **g** TACE activity in endothelial cells treated with solvent (Sol) or CAPN1 (C1; 0.3 U/ml, 15 min); n = 6 different cell batches (Student’s *t* test). **h** EPCR levels in culture medium from endothelial cells treated with solvent (Sol) or CAPN1 (0.3 U/ml, 15 min) in the absence or presence of TAPI; *n* = 7 different cell batches (two-way ANOVA and Sidak’s post-test). **i** Consequence of extracellular CAPN1 (C1; 0.3 U/ml, 4 h) on the surface expression of TNFα, bar = 10 µm; *n* = 6 different cell batches (Student’s *t* test)

To identify potential CAPN1 targets on the endothelial cell surface, cultured human endothelial cells (first passage) were treated with solvent or CAPN1 and the cell supernatant analyzed by mass spectrometry (MS). The calpain concentration used (0.3 U/ml) was chosen to match the calpain activity in plasma from diabetic patients (Online Fig. 1). Several proteins were only detected in samples from CAPN1-treated cells, e.g. plastin-3 (an actin-binding protein) and the endothelial protein C receptor (EPCR), indicating that they are potential CAPN1 targets (Online Table 1). Consistent with these results, the EPCR was detectable in plasma samples from non-diabetic individuals and subjects with type 2 diabetes, but levels were significantly higher in samples from the diabetic group (Fig. 1a). Moreover, there was a positive correlation between plasma calpain activity and EPCR levels (Fig. 1b). Because of these observations and the fact that EPCR can be cleaved from the cell membrane in different pathological conditions [20, 26], we focused on EPCR.

To determine whether calpain plays a causative role in the increase in soluble EPCR in vivo, mice were made diabetic with STZ and treated with either vehicle or the calpain inhibitor N-(1-benzyl-2-carbamoyl-2-oxoethyl)-2-[E-2-(4-diethyl-aminomethylphenyl) ethen-1-yl]benzamide (A-705253; 30 mg/Kg/day) for 12 weeks. As with the human samples, low levels of EPCR were detected in plasma from healthy mice. Diabetes induction, however, resulted in a significant increase in plasma EPCR levels in the vehicle-treated, but not the calpain inhibitor-treated, mice (Fig. 1c). Next, intact endothelial cells were treated with solvent or CAPN1 to assess whether the EPCR was a direct CAPN1 substrate. Consistent with the MS data, western blotting and FACS analysis revealed that the expression of EPCR on the endothelial cell surface was attenuated by CAPN1 (Fig. 1d and e). However, the addition of CAPN1 to whole endothelial cell lysates failed to cleave EPCR, even though the well-characterized CAPN1 target; CD31 [30], was cleaved (Online Fig. 2). Thus, the effect of CAPN1 on EPCR shedding was indirect and not the consequence of a direct proteolytic cleavage (Fig. 1f).

The shedding of the EPCR is known to be regulated by an intracellular signaling-dependent process involving the tumor necrosis factor (TNF)-α-converting enzyme (TACE) [35]. Therefore, the possibility that CAPN1 might activate TACE to elicit secondary EPCR shedding was investigated. A basal TACE activity was detected in endothelial cells and was significantly increased following exposure to CAPN1 (Fig. 1g). More importantly, a TACE inhibitor; N-(R)-[2-(hydroxyaminocarbonyl)methyl]-4-methylpentanoyl-L–t-butyl-alanyl-L-alanine, 2-aminoethyl amide (TAPI), prevented the CAPN1-induced shedding of the EPCR (Fig. 1h). Thus, the effects of CAPN1 on the surface expression of EPCR were secondary to the CAPN1-dependent activation of TACE. Since the activation of TACE should logically lead to the generation of its primary product i.e. TNF-α, the effects of CAPN1 on the production of TNF-α were assessed. CAPN1 treatment resulted in the shedding of TNF-α from the endothelial cell surface confirming CAPN1-mediated TACE activation (Fig. 1i).

### Role of the protease-activated receptor 1 (PAR-1) in the response to extracellular CAPN1

**Fig. 2:**
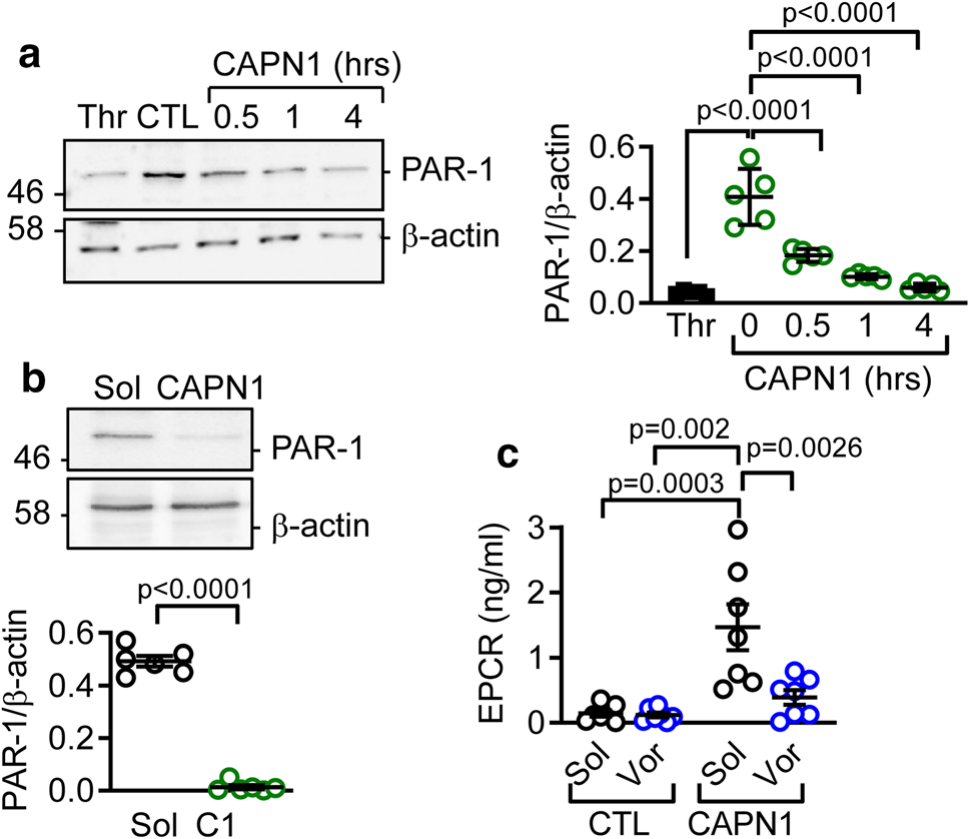
CAPN1 cleaves PAR-1. **a** Comparison of the effects of thrombin (1 U/ml, 15 min) and CAPN1 (0.3 U/ml for up to 4 h) on the surface expression of PAR-1 in human endothelial cells; *n* = 5 different cell batches (one-way ANOVA and Tukey’s post-test). **b** PAR-1 levels in endothelial cell lysates treated with solvent (Sol) or CAPN1 (0.3 U/ml, 30 min); *n* = 6 different cell batches (Student’s *t* test). **c** EPCR levels in culture medium from endothelial cells treated with solvent (Sol) or CAPN1 (0.3 U/ml, 15 min) in the absence or presence of vorapaxar (Vor, 1 µmol/L); *n* = 7 different cell batches (two-way ANOVA and Sidak’s post-test)

Proteases such as thrombin can activate the protease-activated receptor (PAR)-1 to promote the activation of TACE and the subsequent shedding of EPCR [17, 18]. Therefore, we investigated the possibility that CAPN1 might target PAR-1. It was possible to show that CAPN1 induced a time-dependent decrease in the surface expression of the N-terminal domain of PAR-1, which is consistent with receptor activation (Fig. 2a). In this case, treating endothelial cell lysates with CAPN1 also resulted in the proteolytic cleavage of PAR-1, indicating that it was directly cleaved by the protease (Fig. 2b). To identify the potential CAPN1 cleavage site, recombinant PAR-1 was incubated with CAPN1 in vitro and the peptides generated analyzed by MS (Online Table 2). Several N-terminal PAR-1 peptides were detected after CAPN1 treatment and although it was not possible to identify a specific calpain cleavage site, the peptides identified were concentrated around Arg41, which is the canonical thrombin cleavage site [46]. Should CAPN1 act similarly to thrombin, PAR-1 signaling would be expected to link extracellular CAPN1 with EPCR shedding. This was the case as the PAR-1 antagonist; vorapaxar, prevented the CAPN1-induced loss of EPCR from the endothelial cell surface (Fig. 2c). Similarly, an antibody that prevents PAR-1 cleavage and activation [31], also prevented the CAPN1-induced EPCR shedding (Online Fig. 3).

**Fig. 3:**
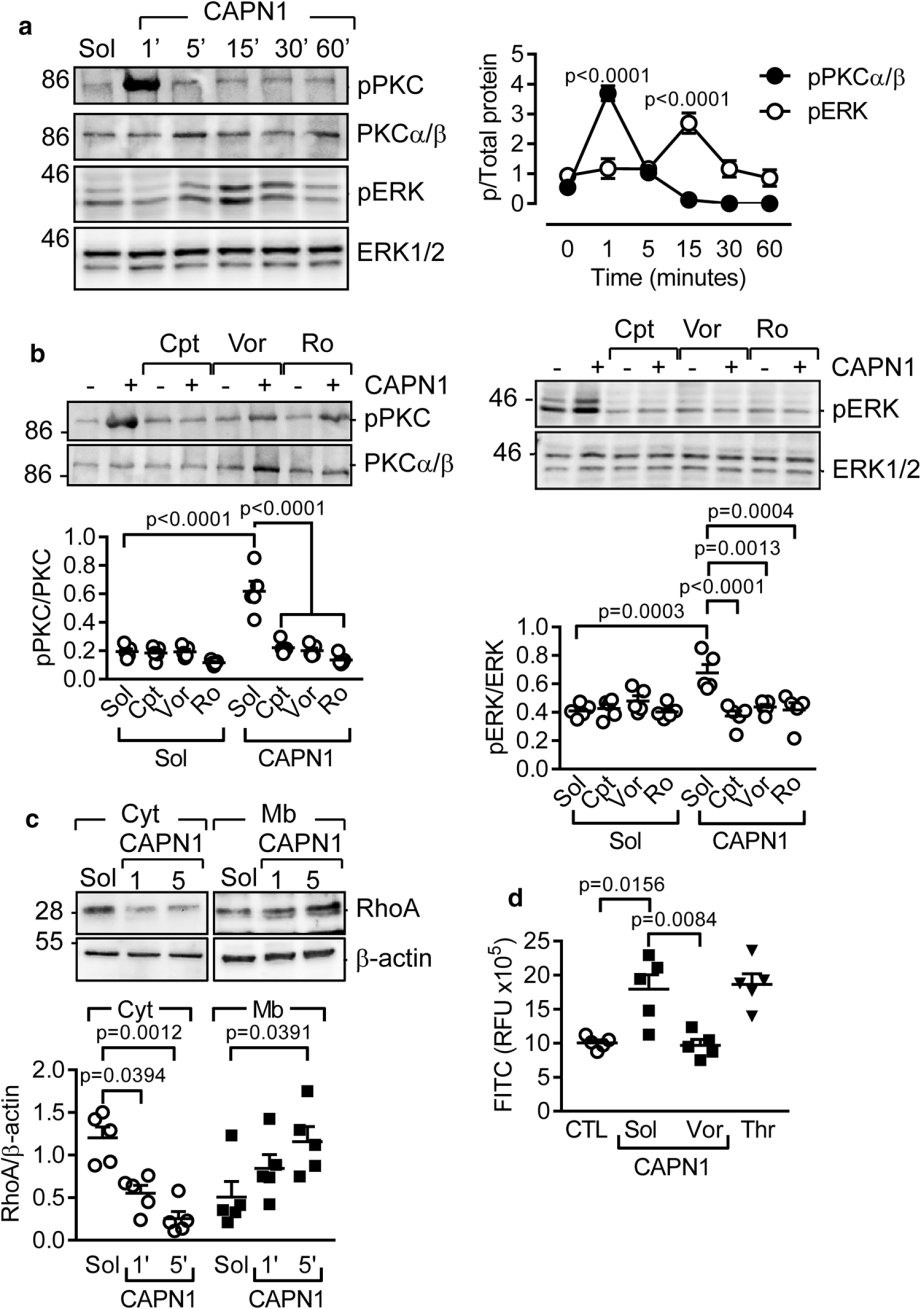
CAPN1 initiates intracellular signaling. **a** Effect of extracellular CAPN1 (0.3 U/ml) on the phosphorylation of PKCα/β (pPKC) and ERK1/2 (pERK) in human endothelial cells; *n* = 7 different cell batches for pERK and *n* = 5 different cell batches for pPKC (two-way ANOVA and Tukey’s post-test). **b** Effect of calpeptin (Cpt, 10 µmol/L), vorapaxar (Vor, 1 µmol/L) and Ro-318220 (Ro, 300 nmol/L) on the CAPN1-induced phosphorylation of PKC (after 1 min) and ERK1/2 (after 15 min); *n* = 5 different cell batches (Two-way ANOVA and Tukey’s post-test). **c** Effect of extracellular CAPN1 (0.3 U/ml, up to 5 min) on the membrane translocation of RhoA in human endothelial cells; *n* = 5 different cell batches (Two-way ANOVA and Tukey’s post-test). **d** Effect of CAPN1 (0.3 U/ml, 15 min) and thrombin (1 U/ml, 15 min) on the permeability of endothelial cells to FITC-dextran (10 kDa). Experiments were performed in the absence and presence of vorapaxar (Vor, 1 µmol/L); *n* = 5 different cell batches (one-way ANOVA and Tukey’s post-test)

Thrombin-induced PAR-1 activation elicits Gαq-dependent signaling to result in increased intracellular Ca^2+^, Rho activation and the phosphorylation of protein kinase (PK) C, extracellular regulated kinase (ERK) 1/2 and AKT [32]. We reasoned, therefore, that should the CAPN1-mediated cleavage of the N-terminus of PAR-1 lead to the generation of a tethered ligand similar to that generated by thrombin, then the addition of CAPN1 to endothelial cells should initiate PAR-1-dependent signaling. Indeed, extracellular CAPN1 elicited a rapid (within 60 s), but subtle increase in intracellular Ca^2+^ that was not observed in cells pretreated with the PAR-1 antagonist (Online Fig. 4). Moreover, CAPN1 increased the phosphorylation of PKCα/β and ERK1/2 (Fig. 3a), without affecting AKT phosphorylation (Online Fig. 5a). The CAPN1-induced phosphorylation of ERK1/2 was prevented by the calpain inhibitor calpeptin as well as by the PAR-1 antagonist, and the PKC inhibitor Ro-318220 (Fig. 3b). A further characteristic consequence of PAR-1 activation in endothelial cells is the activation of RhoA to alter barrier function [3, 36]. CAPN1 also initiated RhoA activation (Fig. 3c), and increased endothelial cell permeability to FITC-dextran to a similar extent as thrombin (Fig. 3d). The latter effect of CAPN1 was also prevented by the PAR-1 antagonist. However, CAPN1 had no effect on Rac1 which has been shown to enhance barrier integrity (Online Fig. 5b). The effects of CAPN1 were also unrelated to non-specific effects on cell viability since neither caspase 3 nor the numbers of early (FITC Annexin V positive and propidium iodide negative) or late (FITC Annexin V and propidium iodide positive) apoptotic cells were altered even at concentrations of up to 1 U/ml for up to 4 h (Online Fig. 6). These findings indicate that extracellular calpains can cleave the PAR-1 receptor to initiate a signaling cascade similar to that activated by thrombin.

### CAPN1-mediated PAR-1 activation on endothelial cells induces adhesion molecule expression and enhances monocyte adhesion

**Fig. 4:**
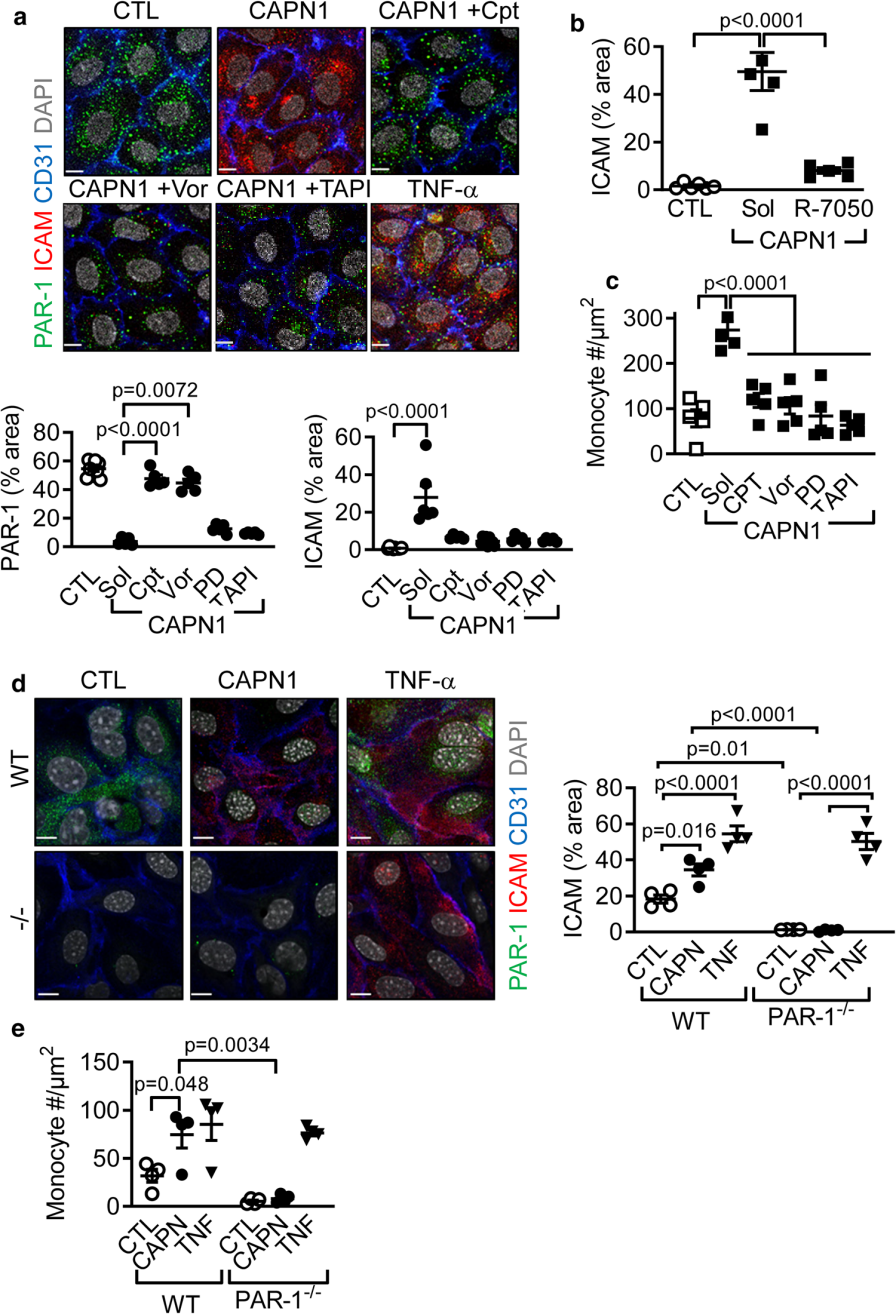
Effect of CAPN1 on the expression of inflammatory markers and monocyte adhesion. **a** Effect of extracellular CAPN1 (0.3 U/ml, 4 h) on the surface expression of PAR-1 (green) and ICAM-1 (red) in human endothelial cells. Experiments were performed in the absence or presence of calpeptin (Cpt, 10 µmol/l), vorapaxar (Vor, 1 µmol/L) and PD98059 (PD, 10 µmol/L). TNF-α was included as positive control; CD31 = blue, DAPI = grey, bar = 10 µm; *n* = 9 different cell batches for CTL and five different cell batches for the CAPN1-treated (one-way ANOVA and Tukey’s post-test). **b** Effect of the TNF-α receptor antagonist; R-7050 (10 µmol/L) on the CAPN1 (0.3 U/ml, 4 h)-induced surface expression of ICAM-1 (red) in human endothelial cells. CD31 = blue, DAPI = grey, bar = 10 µm; *n* = 5 different cell batches (one-way ANOVA and Tukey’s post-test). **c** Monocyte adhesion to endothelial cells treated with solvent (Sol) or CAPN1 (0.3 U/ml, 4 h) in the absence or in the presence of calpeptin (Cpt, 10 µmol/L), vorapaxar (Vor, 1 µmol/L) and PD98059 (PD, 10 µmol/L); *n* = 5 different cell batches (one-way ANOVA and Tukey’s post-test). d Effects of CAPN1 (C1; 0.3 U/ml, 4 h) and TNF-α (10 ng/ml, 4 h) on the surface expression of PAR-1 (green) and ICAM-1 (red) in endothelial cells from wild-type (WT) and PAR-1^−/−^ (−/−) mice; CD31 = blue, DAPI = grey, bar = 10 µm; *n* = 4 different cell batches (two-way ANOVA and Tukey’s post-test). **e** Monocyte adhesion to endothelial cells isolated from wild-type or PAR1^−/−^ mice after stimulation with solvent, CAPN1 (C1; 0.3 U/ml, 4 h) or TNF-α (10 ng/ml, 4 h); *n* = 4 different cell batches (two-way ANOVA and Tukey’s post-test)

Next, the functional consequences of CAPN1-mediated PAR-1 activation on endothelial cells were assessed. TNF-α liberated by TACE can elicit vascular inflammation by inducing the expression of adhesion molecules, such as, intercellular adhesion molecule (ICAM)-1. In endothelial cells cultured under basal conditions, there was no detectable expression of ICAM-1 and N-terminal intact PAR-1 was readily detectable on the endothelial cell surface. Within four hours of CAPN1 addition to the cells, there was a decrease in the N-terminal PAR-1 signal and the induction of ICAM-1 (Fig. 4a). The effects of CAPN1 were comparable with those initiated by TNF-α, and were abrogated in the presence of calpeptin, the PAR-1 antagonist and the TACE inhibitor. A similar effect was observed when TACE was downregulated with small interfering RNAs (Online Fig. 7a and b), confirming the involvement of TACE in CAPN-1-induced ICAM-1 expression**.** Moreover, the CAPN1-induced increase in ICAM-1 expression was sensitive to the TNF-α receptor antagonist; R7050 (Fig. 4b**).** Extracellular CAPN1 also elicited the rapid phosphorylation of the p65 subunit of nuclear factor (NF) κB (Online Fig. 7c), which was also inhibited by R7050 (Online Fig. 7d). Thus, the CAPN1-induced expression of ICAM-1 was secondary to the local liberation of TNF-α.

Consistent with the data on ICAM-1 expression, CAPN1 also increased the adhesion of monocytes to endothelial cells, via a calpeptin-, vorapaxar- and TAPI-sensitive mechanism (Fig. 4c). To further confirm the dependency on PAR-1, experiments were repeated using endothelial cells from wild-type and PAR-1^−/−^ mice. The addition of CAPN1 or TNF-α to cells from wild-type mice resulted in changes similar to those observed in human endothelial cells that were also sensitive to calpeptin, vorapaxar and PD98059 (Fig. 4d and e, Online Fig. 7e). Endothelial cells from PAR-1^−/−^ mice failed to increase ICAM-1 expression following the application of CAPN1, but did respond to TNF-α in a manner similar to the cells from wild-type mice.

### CAPN1 carried by PMPs mediates the effect in vivo

**Fig. 5:**
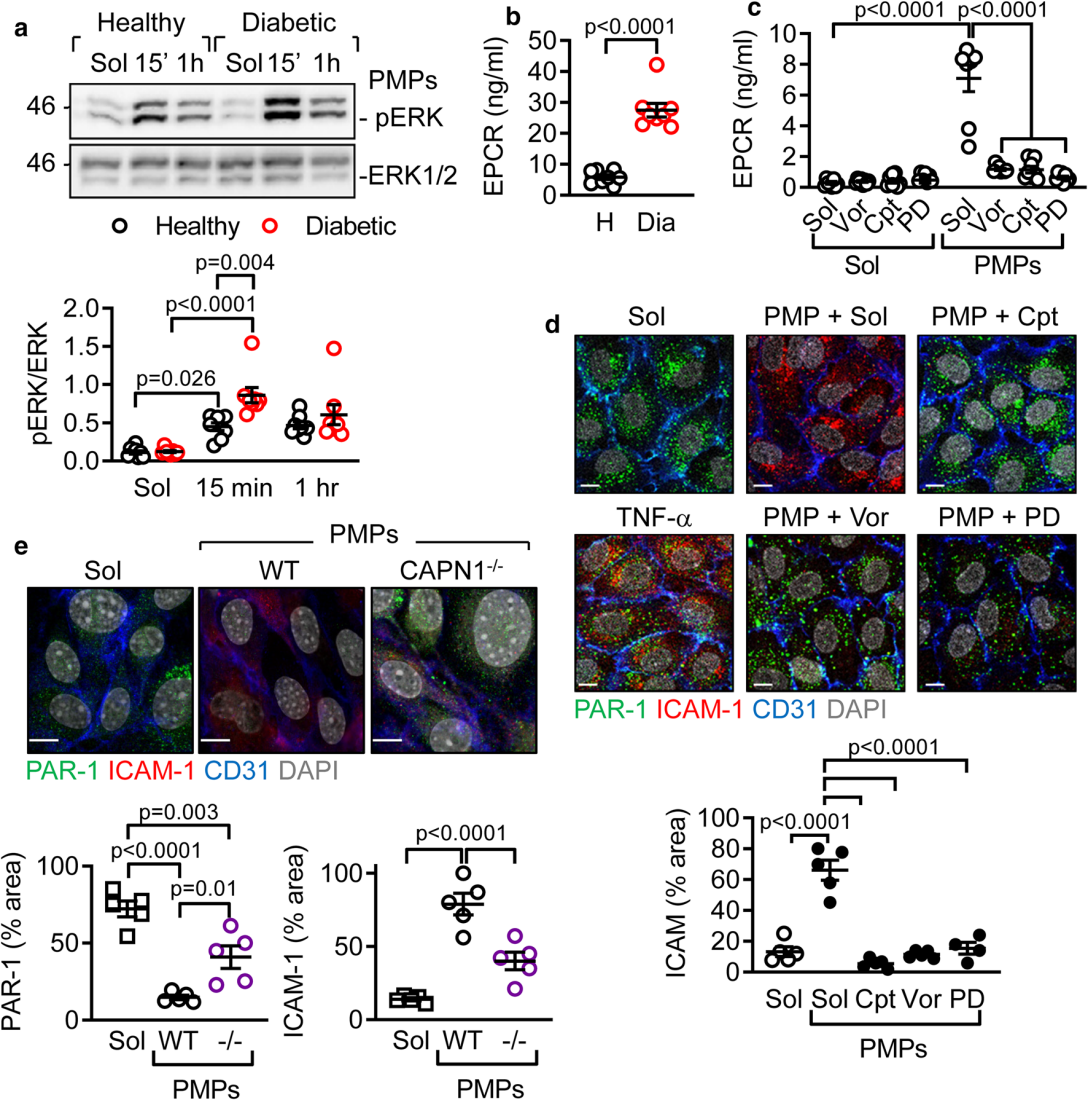
CAPN carried by PMPs initiates intracellular signaling. **a** Phosphorylation of ERK1/2 (pERK1/2) in human endothelial cells treated with solvent (Sol) or PMPs (2 × 10^5^/ml) from healthy or diabetic subjects for 15 min or 1 h; *n* = 8 subjects per group (two-way ANOVA and Tukey’s post-test). **b** EPCR levels in culture medium collected from human endothelial cells treated with PMPs from healthy (H) versus diabetic (Dia) subjects; *n* = 8 subjects per group (Student’s *t* test). **c** EPCR in the endothelial cell supernatant following treatment with PMPs generated from washed human platelets. Experiments were performed in the absence or presence of calpeptin (Cpt, 10 µmol/L), vorapaxar (Vor, 1 µmol/L) and PD98059 (PD, 10 µmol/L); *n* = 8 different cell batches and PMP preparations (two-way ANOVA and Tukey’s post-test). **d** Effect of solvent (Sol), PMPs (2 × 10^5^ /ml, 1 h) and TNF-α on the expression of PAR-1 (green) and ICAM-1 (red) by human endothelial cells. Experiments were performed in the absence or presence of calpeptin (Cpt, 10 µmol/L), vorapaxar (Vor, 1 µmol/L) and PD98059 (PD, 10 µmol/L). CD31 = blue, DAPI = grey, bar = 10 µm; *n* = 5 different cell batches and PMP preparations (one-way ANOVA and Tukey’s post-test). **e** ICAM-1 (red) and PAR-1 (green) expression on the surface of endothelial cells from wild-type mice following treatment with solvent (Sol) or PMPs generated from wild-type (WT) or CAPN1^−/−^ mice. CD31 = blue, DAPI = grey, bar = 10 µm; *n* = 5 different cell batches and PMP preparations (one-way ANOVA and Tukey’s post-test)

Circulating PMPs from diabetic subjects contained higher levels of CAPN1 than those from non-diabetic donors, whereas the levels of CAPN2 were not significantly different between the two groups (Online Fig. 8). To determine whether the CAPN1 carried by PMPs also targeted PAR-1, human endothelial cells were incubated with microparticles isolated from the plasma of healthy volunteers or individuals with type 2 diabetes. Microparticles from healthy individuals elicited the phosphorylation of endothelial cell ERK1/2 (Fig. 5a), and stimulated EPCR shedding (Fig. 5b), but the effects were clearly more pronounced when microparticles from diabetic patients were used. Although the majority of circulating microparticles are known to be PMPs, the involvement of PMPs in mediating these effects was further confirmed using washed human platelet-derived microparticles. Indeed, in vitro generated PMPs also elicited EPCR shedding (Fig. 5c), and increased ICAM-1 expression on human endothelial cells (Fig. 5d). Both effects were inhibited by pre-incubation of the PMPs with calpeptin or by treating endothelial cells with either the PAR-1 antagonist or the MEK inhibitor. A similar approach using platelets from wild-type mice gave identical results (Fig. 5e); however PMPs from CAPN1^−/−^ mice elicited much weaker effects. The residual effects observed are most likely attributable to CAPN2 which is also activated by the ionomycin used to generate murine PMPs [37].

### Specific deletion of CAPN1 in platelets protects mice from diabetes-associated vascular inflammation

**Fig. 6:**
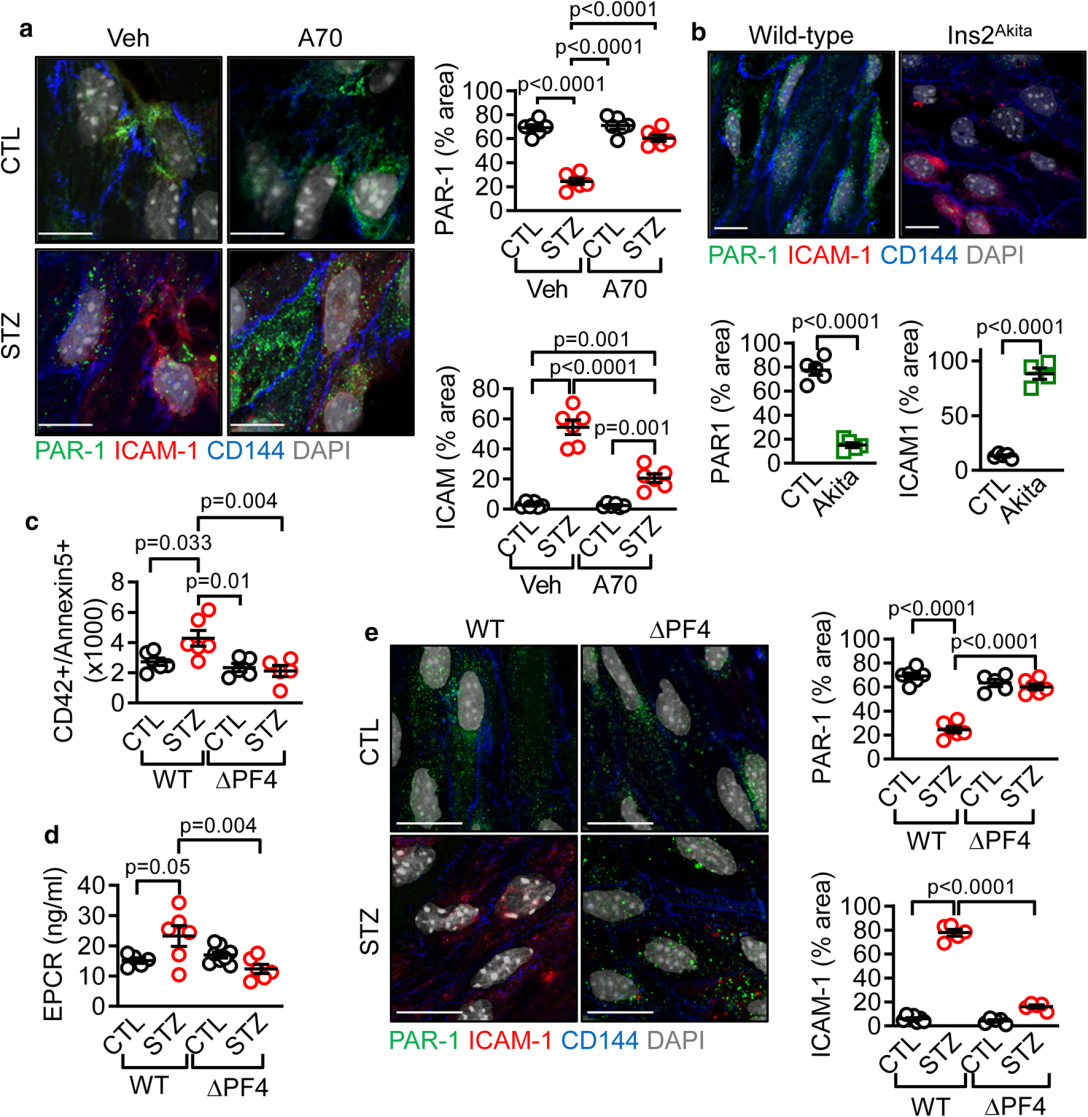
Effect of calpain inhibition and platelet-specific CAPN1 deletion on diabetes-induced vascular inflammation. **a** PAR-1 (green) and ICAM-1 (red) expression in *en face* preparations of aortae from wild-type mice given saline (CTL) or made diabetic with streptozocin (STZ) and treated with either vehicle (Veh) or the calpain inhibitor A705232 (A70, 30 mg/kg/day) for 12 weeks; bar = 10 µm, *n* = 6 animals per group (one-way ANOVA and Tukey’s post-test). **b** PAR-1 (green) and ICAM-1 (red) expression in en face preparations of aortae from 8-month-old non-diabetic wild-type mice or their diabetic Ins2^Akita^ mice littermates. CD144 = blue, DAPI = grey, bar = 10 µm; *n* = 5 mice per group (Student’s *t* test). **c** Circulating PMPs in plasma from wild type (WT) and CAPN1^ΔPF4^ (ΔPF4) mice treated with vehicle (CTL) or made diabetic with streptozocin (STZ) and maintained for 8 weeks; *n* = 5 animals in the ΔPF4 group and six animals in the WT group (one-way ANOVA and Tukey’s post-test). **d** Levels of EPCR in plasma and **e** PAR-1 (green) and ICAM-1 (red) in *en face* preparations of aortae from the same animals as in c; CD144 = blue, DAPI = grey, bar = 10 µm; *n* = 5 animals in the CTL-ΔPF4 group and *n* = 6 animals in the other groups (one-way ANOVA and Tukey’s post-test)

To confirm the link between platelet-derived CAPN1 and the vascular inflammation associated with diabetes in vivo, diabetes was induced in mice using STZ. While aortic endothelial cells from non-diabetic mice clearly expressed PAR-1 with no detectable ICAM-1 expression, the situation was reversed in arteries from the STZ-treated mice, as ICAM-1 was strongly expressed while little or no N-terminal PAR-1 was detectable (Fig. 6a). Treating the animals with the calpain inhibitor, A705232, protected against the diabetes-induced expression of ICAM-1 and prevented the loss of PAR-1 in aortic endothelial cells in situ. Importantly, the loss of PAR-1 and the increase in ICAM-1 were not limited to STZ-induced diabetes as aortic endothelial cells from Ins2^Akita^ diabetic mice displayed a similar phenotype (Fig. 6b).

To demonstrate the role played by platelet-derived CAPN1 in this process, floxed CAPN1 mice were crossed with PF4-deleter mice to generate animals lacking CAPN1 specifically in platelets, so called CAPN1^ΔPF4^ mice. Compared to their wild-type littermates, plasma from CAPN1^ΔPF4^ mice contained low levels of PMPs and the induction of diabetes significantly increased plasma PMP numbers in wild-type but not in CAPN1^ΔPF4^ mice (Fig. 6c). Similarly, plasma EPCR levels were enhanced by diabetes in wild type, but not in CAPN1^ΔPF4^ mice (Fig. 6d). In animals made diabetic using a high-fat diet (20 weeks), significant numbers of monocytes adhered (ex vivo) to the aortic endothelium (Online Fig. 9). Significantly fewer monocytes adhered to the endothelium of CAPN1^ΔPF4^ mice. The CAPN1^ΔPF4^ mice were also protected from diabetes-induced vascular inflammation as while STZ-treatment resulted in a decrease in PAR-1 and an increase in ICAM-1 expression in aortic endothelial cells from wild-type mice no such changes were observed in aortae from mice lacking platelet CAPN1 (Fig. 6e).

## Discussion

The results of this study indicate that platelet-derived CAPN1, carried by PMPs, cleaves PAR-1 on endothelial cells to initiate a cascade of events leading to the activation of TACE and the shedding of EPCR and TNF-α. The latter increases the expression of adhesion molecules and promotes vascular inflammation. It was possible to demonstrate correlative changes in circulating EPCR levels in plasma from healthy and diabetic subjects as well as non-diabetic and diabetic mice. Also in mice, calpain inhibition and the platelet-specific deletion of CAPN1 both prevented the diabetes-induced increase in circulating EPCR as well as the associated vascular inflammation. These data highlight a novel mechanism by which activated platelets can directly affect the homeostasis of the vascular wall to initiate the vascular complications associated with diabetes and vascular disease (see Online Fig. 10).

Calpains are involved in a variety of Ca^2+^-regulated cellular processes by inducing the partial proteolysis of a broad spectrum of substrates [16]. Although these effects have been mainly attributed to the activation of intracellular calpains, the proteases are also found in the circulation [14, 34], where they have been linked with angiogenesis and vascular repair, in part by cleaving fibronectin and amplifying the effects of vascular endothelial growth factor [27]. In the present study, CAPN1 carried by PMPs was found to induce the shedding of EPCR from the vascular wall. EPCR is a type I transmembrane protein that is important for the generation of the potent anticoagulant and cytoprotective protein; activated protein C (APC) [13, 43]. While APC inactivates factors Va and VIIIa to exert its anticoagulant effects, its cell-protective actions are attributed to the cleavage of PAR-1 [41], an effect that requires its binding to EPCR [29, 40, 41]. This explains why EPCR has been generally classified as cytoprotective and why EPCR shedding has been associated with vasculopathy [26, 42]. Although the EPCR was picked up by the proteomic approach used to identify endothelial cell surface proteins targeted by CAPN1, the protease was unable to cleave the EPCR in cell lysates, indicating that it was an indirect target. Rather, fitting with the fact that the shedding of the EPCR is controlled by TACE [18, 35], it was possible to prevent the CAPN1-induced decrease in EPCR using a TACE inhibitor. Ours is not the first report to link calpain with the regulation of TACE as the Ca^2+^ ionophore-induced, TACE-dependent shedding of glycoprotein Ibα in platelets was previously attributed to calpain activation [47]. However, the authors of the latter study did not address the mechanisms involved or the physiological consequences in any detail. Perhaps the best known target of TACE is TNF-α [28], which is synthesized as a 26 kDa transmembrane pro-protein that is bound to the endothelial cell surface. TNF-α released as a 17 kDa peptide into the extracellular space only after TACE activation. We found that TNF-α was present on the endothelial cell surface and that extracellular CAPN1 effectively decreased the cell-bound form of the protein. The consequence of this process was endothelial cell activation and the expression of ICAM-1. Such an increase in the levels of adhesion molecules on the surface of endothelial cells is a prerequisite for the adhesion of circulating monocytes and represents an early step in the development of inflammatory responses.

The next step was to identify a link between extracellular CAPN1 and the activation of a signaling cascade that could affect TACE activation. We focused on PAR-1, as this receptor has been previously linked with TACE-dependent EPCR shedding [17]. Moreover, the activation of PAR-1 requires the proteolytic cleavage of the extracellular N-terminal domain of the protein, thus generating an amino terminus that functions as a tethered ligand to initiate signaling [1]. CAPN1 was found to cleave the PAR1 receptor and generate PAR-1-derived peptides similar to those generated by thrombin, which cleaves the N-terminal domain of the protein at Arg41 [46]. That a protease other than thrombin is able to activate the PAR-1 receptor is not that unusual, as other proteases can cleave the receptor, albeit at distinct sites. For example, the APC/EPCR complex can cleave PAR-1 at Arg41 and Arg46 [40], while matrix metalloprotease-1 cleaves PAR-1 to create a longer tethered ligand [23]. The different proteases activate different signaling pathways, depending on the ligands released. While thrombin-induced PAR-1 activation leads to Gq-dependent signaling resulting in increased intracellular Ca^2+^, Rho activation as well as the phosphorylation of protein kinase C, ERK1/2 and AKT [32], APC-induced signaling involves Gi proteins, Rac and even transactivation of the sphingosine 1-phosphate receptor [8, 9]. We found that the CAPN1-induced signaling via the PAR-1 receptor resulted in the activation of ERK1/2 and RhoA but not AKT. Like thrombin, CAPN1 also disrupted endothelial cell barrier function to increase permeability as well as TACE activity. Such a mechanism very probably contributes to the diabetes-associated vascular leakage that characterizes diabetic microangiopathy.

Where does extracellular CAPN1 come from? CAPN1 has been identified in the platelet secretome [11], but most circulating CAPN1 in diabetic individuals is contained in PMPs. Such microparticles are an effective way to transport and transfer biological information as they can fuse with the membrane of target cells to deliver their contents at the cell surface or can be internalized through presentation of specific antigens [10, 25]. Although recombinant CAPN1 was used in many of the experiments performed in the present study, the effects of CAPN1 could be reproduced by microparticles generated in vitro from washed platelets. Given that platelets contain CAPN1 and CAPN2 [24], and that PMPs represent up to 80% of all circulating microparticles [10, 19], it seems reasonable to assume that PMPs are the main source of circulating calpains. However, not all PMPs are able to cleave PAR-1, possibly because not all PMPs carry high amounts of activated calpains. Importantly, we could show that microparticles isolated from healthy individuals had minimal effects on EPCR shedding while microparticles from diabetic subjects (which contain more active CAPN1) elicited a pronounced effect. In the present study, ionomycin was used to generate murine PMPs in vitro, a stimulus known to activate both CAPN1 and CAPN2 [15, 37]. However, it was possible to demonstrate that PMPs from CAPN1^−/−^ mice were less effective at decreasing PAR-1 levels and increasing endothelial cell ICAM-1 expression than PMPs from wild-type mice. To demonstrate the in vivo relevance of the pathway described, mice were made diabetic with STZ. While the induction of diabetes led to the loss of the N-terminal of the PAR-1 receptor on aortic endothelial cells and a concomitant induction of ICAM-1, the animals given the calpain inhibitor were protected. Some authors have expressed concern about the use of STZ to induce diabetes [5], but we were able to confirm our findings in diabetic (type I) Ins2^Akita^ mice which carry a mutation in the Ins2 gene [48, 49]. Moreover, we have previously reported that calpain activation in platelets was found in both type 1 and type 2 diabetes [37].

The calpain inhibitor used was previously reported to prevent platelet activation in diabetic mice [37], as well as to prevent the diabetes-induced generation of platelet microRNAs that could potentially affect endothelial cell protein expression [7]. Therefore, diabetes-induced changes in PAR-1 and ICAM-1 expression were studied in animals specifically lacking CAPN1 in platelets. The importance of platelet-derived CAPN1 in the vascular complications of diabetes was demonstrated by the fact that diabetes induction in CAPN1^ΔPF4^ mice failed to increase circulating levels of the EPCR or to alter the surface expression of PAR-1 or ICAM-1 on endothelial cells. Our findings have a clear pathophysiological relevance as they imply that increased circulating levels of calpain-enriched PMPs in diabetic patients are responsible for the activation of the endothelial cell PAR-1 receptor. This in turn can initiate a sterile vascular inflammation via the release of TNF-α, to promote EPCR shedding and thus attenuate cytoprotective EPCR-APC signaling. As CAPN1 is ubiquitously expressed, it is clear that the activation of the protease in endothelial cells can also contribute to vascular dysfunction and the activation of endothelial cell CAPN1 was reported to cleave prostaglandin synthase in small arteries to decrease prostacyclin formation [38]. However, the finding that platelet-derived calpain carried by PMPs circulates has important implications for the development of vascular disease, and can contribute to the spread of endothelial cell activation and vascular inflammation.

## Electronic supplementary material

Below is the link to the electronic supplementary material.

Supplementary file1 (XLSX 76 KB)

Supplementary file2 (XLSX 25 KB)

Supplementary file3 (PDF 1667 KB)
