# Supplementary material for: Platelet-derived calpain cleaves the endothelial protease-activated receptor 1 to induce vascular inflammation in diabetes
Source: Basic Res Cardiol. 2020 Dec 1;115(6):75. doi: 10.1007/s00395-020-00833-9 (PMC7716944; doi:10.1007/s00395-020-00833-9)
Supplement: Supplementary file 3 — Supplementary file3 (PDF 1667 KB) [file 395_2020_833_MOESM3_ESM.pdf]

SUPPLEMENTAL MATERIAL

Platelet-derived calpain cleaves the endothelial protease-activated receptor 1 to induce vascular inflammation in diabetes

Anastasia Kyselova,[1,2] Amro Elgheznawy,[1] Ilka Wittig,[2,3] Juliana Heidler,[2,3] Alexander W. Mann,[4] Wolfram Ruf,[2,5,6] Ingrid Fleming,[1,2] Voahanginirina Randriamboavonjy.[1,2]

[1]Institute for Vascular Signalling, Centre of Molecular Medicine, Goethe University, Frankfurt am Main, D-60596, Germany.

[2]German Center for Cardiovascular Research (DZHK), Partner site Rhein-Main, Frankfurt am Main, Germany.

[3]Functional Proteomics, SFB 815 Core Unit, Goethe University, Frankfurt am Main, Germany.

[4]Endokrinologikum Frankfurt, Frankfurt am Main, D-60596, Germany.

[5]Center for Thrombosis and Hemostasis, University Medical Center, Mainz, Germany.

[6]Department of Immunology and Microbiology, the Scripps Research Institute, La Jolla, CA, USA.

Short title: Calpain-mediated activation of PAR-1 in diabetes

Text word count: 7557

Address correspondence to: Voahanginirina Randriamboavonjy PhD, Institute for Vascular Signalling, Centre of Molecular Medicine, Goethe-University, Theodor-Stern-Kai 7, 60596 Frankfurt am Main, Germany. Tel: (+49) 69 6301 6973; Fax: (+49) 69 6301 86880; Email: voahangy@vrc.uni-frankfurt.de.

Materials

Cell culture media were purchased from Gibco (Invitrogen; Darmstadt, Germany). Calpeptin was from Calbiochem (Darmstadt, Germany), thrombin was Hemochrom Diagnostica (Essen, Germany), type I Collagen and fibronectin were from BD Transduction Laboratories (Heidelberg, Germany). FITC-dextran was from Thermo Scientific (Dreieich, Germany), the human recombinant PAR-1 was from Abnova (Heidelberg, Germany) and the TNFα receptor antagonist R-7050 was from Santa Cruz Biotechnology (Heidelberg, Germany), Lipofectamine RNAiMax was from Invitrogen (Darmstadt, Germany). The calpain inhibitor A-705253 [5], was provided by Abbvie (Ludwigshafen, Germany). Recombinant calpain 1 and all other products were purchased from Sigma-Aldrich (Taufkirchen, Germany).

Antibodies

The anti-PKCα/β (#61010, #61012) antibody was from BD transduction laboratories (Heidelberg, Germany). The anti-Rac1 (#PA1-091) antibody and secondary alexa fluor conjugated antibodies (#A21202, #A21206, #A10036, #A10040, #A11056, #A21447, #A31573, #A31571) were purchased from Thermo Scientific (Dreieich, Germany). The anti-Akt (#9272); anti-Ser^{473}-Akt (#4058), anti-ERK (#9102), anti-Thr^{202}/Tyr^{204}ERK (#9101), anti-Thr$^{638/641}$PKCα/β (#9375) antibodies were from Cell Signalling (New England Biolabs, Frankfurt, Germany). The anti-PAR-1 (#5605), the anti-PAR1 ATAP-2 (#13503), anti-EPCR (#53982), anti-ICAM-1 (#8439), anti-RhoA (#179), anti-TACE (#3973) and anti-VE-cadherin (#6458) antibodies were from Santa Cruz Biotechnology (Heidelberg, Germany). The human anti-PAR-1 (#32611) antibody was from Abcam (Cambridge, UK).

Platelets and microparticles isolation

Human platelets: Platelets were obtained by centrifugation (900g, 7 minutes) of platelet-rich plasma, as described [6]. The resulting pellet was washed in Ca^{2+}-free HEPES buffer (mmol/L:

$NaCl$, 136; KCl, 2.6; $MgCl_2$, 0.93; NaH_2PO_4, 3.26; glucose, 5.5; HEPES, 3.7; pH 7.4 at 37°C) and samples were re-suspended in HEPES buffer.

Murine platelets: Mice were anesthetized with isoflurane and blood was collected via cardiac puncture into a syringe containing 10% acidic citrate dextrose (120 mmol/L sodium citrate, 110 mmol/L glucose, 80 mmol/L citric acid) as anticoagulant. Platelets were prepared from whole blood by differential centrifugation and resuspended in HEPES buffer.

Isolation of circulating microparticles

Platelet poor plasma was centrifuged at 20,000g for 45 minutes at 4°C. Microparticle pellets were washed, re-suspended in PBS and subjected to three rounds of washes in PBS. After final centrifugation to remove contaminants, microparticles were re-suspended in PBS or cell culture medium for further use.

Generation of platelet-derived microparticles

Human or murine platelet suspensions were stimulated with Ca^{2+} (2 mmol/L) and ionomycin (1 µmol/L) for 1 hour. The suspension was centrifuged at 900g for 5 minutes to discard intact platelets and the resulting supernatant was centrifuged at 20000g for 45 minutes to concentrate microparticles. Microparticles were counted using a BD FACSVerse flow cytometer (BD Biosciences, Heidelberg, Germany). The MP-gate was determined using Megamix beads (BioCytex, Marseille, France), which is a mix of three beads with diameters of 0.5 µm, 0.9 µm and 3 µm, respectively. MPs were defined as particles less than 1.0 µm in size. PMPs were defined as MP positive for Annexin V (#556422) and CD42 (#555472) purchased from BD Pharmingen (Heidelberg, Germany). Calpain carried by MP was labelled using anti-CAPN1 (#7531) or anti-CAPN2 antibody (#7533) from Santa Cruz, Heidelberg, Germany). The secondary alexa fluor conjugated antibody (#A-21447) was from Thermo Scientific (Dreieich, Germany).

Cell culture

Human umbilical vein endothelial cells were isolated and cultured as described [1] and confluent cells up to passage 2 were used for the different experiments. Murine lung endothelial cells (MLEC) were isolated from either wild-type or $PAR1^{-/-}$ mice and cultured as described [3] and cells were used up to passage 8. THP-1 monocytic cells were cultured in RPMI 1640 containing 100 units/ml penicillin, 100 µg/ml streptomycin and 10% heat inactivated fetal calf serum. The use of human material in this study conforms to the principles outlined in the Declaration of Helsinki and the isolation of endothelial cells was approved in written form by the ethics committee of the Goethe-University.

Isolation of murine monocytes

Murine whole blood was centrifuged (150g, 20 minutes) and platelet-rich plasma was discarded. Buffy coat was then centrifuged on Biocoll gradient solution (density 1.077; Biochrom, Berlin, Germany) at 400 g for 30 minutes at room temperature. The major band, containing the mononuclear cells, was harvested, and the mononuclear cells were washed by centrifugation three times and re-suspended in RPMI 1640 supplemented with 5% heat inactivated human serum, 100 units/ml penicillin and 100 µg/ml streptomycin.

Cell adhesion

Human or murine endothelial cells were starved overnight and then incubated with the different inhibitors for 30 minutes prior to stimulation with either TNFα (10 ng/ml, 4 hours) or CAPN1 (0.3U/ml, 4 hours). After the stimulation, THP-1 (500.000) or murine monocytes (150.000) were added to human or murine endothelial cells, respectively. After 30 minutes incubation, non-adherent cells were removed by washing and adherent cells were counted.

FACS analysis

Human endothelial cells were treated with solvent or CAPN1 (0.3 U/ml, 4 hours). After washing, cells were re-suspended in PBS and labeled with either a FITC-conjugated anti-human EPCR (#557950) antibody or a FITC-conjugated IgG1κ isotype control (#555748) (BD Pharmingen).

The suspension was washed, re-suspended in PBS and analyzed in a FACscan flow cytometer using CellQuest software (Becton Dickinson, CA).

Permeability assay

Cell permeability was assessed using a modified Transwell chamber system with membrane inserts (1µm pore size; BD Biosciences, Heidelberg, Germany). Human endothelial cells were seeded at a density of 100.000 cells/cm^2 on the Transwell inserts and subjected to different treatment. After washing, FITC-dextran (10 kDa) was added on the upper part of the well. Permeability was evaluated by counting the fluorescence in the lower wells after different time points.

Assay of calpain activity in plasma

Plasma (50 µL) was diluted in calpain reaction buffer (mmol/L: NaCl, 136; KCl, 2.6; $MgCl_2$, 0.93; NaH_2PO_4, 3.26; glucose, 5.5; HEPES, 3.7; pH 7.4 at 37°C) in the absence or in the presence of the calpain inhibitor calpeptin (50 µmol/L). The calpain substrate N-succinyl-Leu-Leu-Val-Tyr7-amido-4-methylcoumarin (200 µmol/L) was added and the formation of the fluorescent metabolite 7-amino-4-methylcoumarin (AMC) was monitored using a spectrofluorimeter (excitation λ360 nm, emission λ460 nm). Calpain activity corresponds to the difference between OD measured with and without calpeptin and is expressed as AMC concentration.

Immunoblotting

Cells were lysed in ice-cold RIPA buffer (50 mmol/L Tris HCl (pH 7.5), 150 mmol/L NaCl, 25 mmol/L NaF,10 mmol/L $Na_4P_2O_7$,1% Triton X-100, 0,1% SDS and 0.5% sodium deoxycholate).Total proteins were separated by sodium dodecyl sulfate-polyacrylamide gel electrophoresis (SDS-PAGE), subjected to Western Blotting and visualized by enhanced chemiluminescence using a commercially available kit (Amersham, Freiburg, Germany), as described [4]. In some experiments, cell lysates were treated with recombinant CAPN1 (0.3U/ml, 30 °C, 30 minutes) prior to SDS-PAGE. To assess protein phosphorylation, either equal amounts of protein from each sample were loaded twice and one membrane incubated with the phospho-specific antibody and the other with an antibody recognizing total protein, or blots were reprobed with the appropriate antibody. For RhoA and Rac-1 experiments, cells were lysed in ice-cold TritonX-100 buffer and triton soluble and insoluble proteins were separated by SDS-PAGE and subjected to Western Blotting.

Immunostaining

Immunohistochemistry: Human endothelial cells or MLEC were stimulated with either recombinant CAPN1 or platelet-derived microparticles. After washing, the cells were fixed with paraformaldehyde (2% v/v in PBS) and blotted with anti- ICAM-1 antibody (#BBA17)(R&D Systems, Wiesbaden, Germany), anti-CD31 (#M0823, Dako Agilent, Santa Clara, CA United States), anti-PAR1 antibody (#32611, Abcam ,Cambridge, UK), anti-TNFα antibody (#sc-133192, Santa Cruz Biotechnology, Heidelberg, Germany) or IgG control overnight at 4°C followed by fluorescent secondary antibody. Fluorescent images were obtained using a Zeiss LSM-510 Meta laser confocal microscope.

En face staining: Mouse aorta was carefully cleaned of fat and connective tissue. Arteries were opened, washed and fixed with paraformaldehyde (2% v/v in PBS). After washing, arteries were labeled with anti-ICAM antibody (#8439, Santa Cruz Biotechnology, Heidelberg, Germany), anti-CD144 (#6458, Santa Cruz Biotechnology, Heidelberg, Germany) anti-PAR1 antibody (#5605, Santa Cruz Biotechnology, Heidelberg, Germany), or IgG control overnight at 4°C. After staining with fluorescent secondary antibody, arteries were mounted on glass slides with fluorescent mounting medium (Dako, Eching, Germany). *En face* images were obtained using a Zeiss LSM-510 Meta laser confocal microscope.

Measurement of soluble EPCR

EPCR levels were measured in murine or human plasma or in culture media using a commercially available ELISA (Biozol, Eching, Germany), according to the manufacturers protocol.

Measurement of TACE activity

TACE activity was measured in cell lysate using a fluorimetric sensolyte activity assay kit (Anaspec; Eurogentec, Seraing, Belgium).

TACE downregulation

A small interfering RNA (siRNA) approach was used to downregulate TACE expression (s13718, Ambion, Darmstadt, Germany) versus a scrambled siRNA probe (Ambion) as control. Endothelial cells were transfected using lipofectamine RNAiMax (Invitrogen, Darmstadt, Germany) according to the manufacturer's protocol and maintained in culture for 72 hours before stimulation with CAPN1.

Monocyte adhesion to aortic endothelial cells ex vivo

Bone marrow cells were was isolated by flushing femurs and tibias of 8 week-old wild-type mice with PBS, followed by filtering through 70 µm nylon web and centrifugation at 300g for 5 minutes. The supernatant was discarded and cells were lysed on ice with erythrocytes lysis buffer for 5 minutes and centrifuged at 300g for 5 minutes at 4°C. Thereafter, cells were resuspended in PBS and Ficoll solution, centrifuged (800g, 20 minutes, without brake) and the monocyte layer was separated and washed with PBS. Monocyte numbers was determined using a cell counter (CASY; OMNI Life Science, Bremen, Germany). Thereafter monocytes were labeled with MitoSox (Invitrogen, Darmstadt, Germany).

Aortae were isolated from wild-type and CAPN1$^{\Delta PF4}$ mice fed a high fat diet for 20 weeks. Then, aortae were opened and pinned onto sterile agar with the endothelial cell surface upwards in serum free RPMI1640 medium. All aortae were incubated with 1x106 freshly isolated and fluorescently-labeled monocytes for 30 minutes at 37°C. Unbound monocytes were rinsed away and aortas were fixed with 4% PFA and mounted. Monocytes that remained attached to the aortic endothelial cells were counted in 3 consecutive fields using a Zeiss LSM-510 confocal microscope (Carl Zeiss; Jena, Germany).

Proteomics

Collection of culture medium: Culture medium (serum and phenol red-free) from unstimulated and CAPN1-stimulated endothelial cells were collected and proteins were precipitated using 20% w/v trichloric acid for 30 minutes on ice and stored frozen at -20°C overnight. Samples were centrifuged for 30 minutes at 10,000 g and protein pellets were washed with ice cold acetone. Proteins were resolved in 50 µl of 8 M Urea, 50 mmol/L Tris/HCl, ph 8.5, 10 mmol/L DTT and initial incubated for 30 minutes at 22°C. Reduced thiols were alkylated using 40 mmol/L chloroacetamide and the mixture was diluted with four volume of dilution buffer (25 mmol/L Tris/HCl, pH 8.5, 10% acetonitrile). Proteins were digested by adding 10 µl trypsin (0.1 µg/µl) overnight. Samples were acidified with 3µl 10% trifluoroacetic acid and peptides were purified using C18 Stage tips [7].

In vitro protein cleavage with calpain: Recombinant PAR-1 (1µg) was treated *in vitro* with either solvent or recombinant CAPN1 (1U) in HEPES buffer. Samples were diluted in 50mmol/L Ammonium bicarbonate and transferred to 30 kDa cut off filters to collect peptides in the flow through. Peptides were purified by C18-stop and go extraction tips [8] and analyzed by mass spectrometry.

Mass spectrometry analysis: Liquid chromatography / mass spectrometry (LC/MS) was performed on Thermo Scientific™ Q Exactive Plus equipped with an ultra-high performance liquid chromatography unit (Thermo Scientific Dionex Ultimate 3000) and a Nanospray Flex Ion-Source (Thermo Scientific). Peptides were loaded on a C18 reversed-phase precolumn (Thermo Scientific) followed by separation on a 2.4 µm Reprosil C18 resin (Dr. Maisch GmbH)

in-house packed picotip emitter tip (diameter 100 µm, 15 cm long from New Objectives). For endothelial cell samples, peptides were separated on a gradient from mobile phase A (4% acetonitrile, 0.1% formic acid) to 50 % mobile phase B (80% acetonitrile, 0.1% formic acid) for 30 minutes with a flow rate 400 nl/min. For recombinant protein samples, the gradient was from 5% B to 60% B for 90 min.

MS data were recorded by data dependent acquisition Top10 method selecting the most abundant precursor ions in positive mode for HCD fragmentation. The full MS scan range was 300 to 2000 m/z with resolution of 70000, and an automatic gain control (AGC) value of $3x10^6$ total ion counts with a maximal ion injection time of 160 ms. Only positive charged ions were selected for MS/MS scans with a resolution of 17500, an isolation window of 2 m/z and an automatic gain control value set to 10^5 ions with a maximal ion injection time of 150 ms.

MS data were analyzed using MaxQuant 1.5.2.8 [2], Perseus 1.5.4.1 [9], and Excel (Microsoft Office 2013). N-terminal acetylation (+42.01) and oxidation of methionine (+15.99) were selected as variable modifications and carbamido methylation (+57.02) on cysteines as a fixed modification. The human reference proteome set (Uniprot, April 2015, 68506 entries) was used to identify semitryptic peptides and proteins with a false discovery rate (FDR) less than 1%. Reverse identifications and common contaminants were removed from the data set. Gene Ontology (GO) terms were loaded for each identified human protein and filtered for “plasma membrane part”.

MS Data of CAPN1-treated recombinant PAR-1 was analyzed by Peaks Studio 7.5 software. Data were analyzed without setting enzyme specificity, parent mass error tolerance was 10 ppm and fragment error tolerance was 0.05 Da. De novo sequenced peptides were identified using human reference proteome set (68,506 entries, download from Uniprot April/2015) with a false discovery rate <0.01.

References

1. Busse R, Lamontagne D (1991) Endothelium-derived bradykinin is responsible for the increase in calcium produced by angiotensin-converting enzyme inhibitors in human endothelial cells. Naunyn Schmiedebergs Arch Pharmacol 344:126-129.
2. Cox J, Mann M (2008) MaxQuant enables high peptide identification rates, individualized p.p.b.-range mass accuracies and proteome-wide protein quantification. Nat Biotechnol 26:1367-1372.
3. Fleming I, Fisslthaler B, Dixit M, Busse R (2005) Role of PECAM-1 in the shear-stress-induced activation of Akt and the endothelial nitric oxide synthase (eNOS) in endothelial cells. J Cell Sci 118:4103-4111.
4. Fleming I, Fisslthaler B, Dixit M, Busse R (2005) Role of PECAM-1 in the shear-stress-induced activation of Akt and the endothelial nitric oxide synthase (eNOS) in endothelial cells. J Cell Sci 118:4103-4111.
5. Lubisch W, Beckenbach E, Bopp S, Hofmann HP, Kartal A, Kastel C, Lindner T, Metz-Garrecht M, Reeb J, Regner F, Vierling M, Moller A (2003) Benzoylalanine-derived ketoamides carrying vinylbenzyl amino residues: discovery of potent water-soluble calpain inhibitors with oral bioavailability. J Med Chem 46:2404-2412.
6. Randriamboavonjy V, Schrader J, Busse R, Fleming I (2004) Insulin induces the release of vasodilator compounds from platelets by a nitric oxide-G kinase-VAMP-3-dependent pathway. J Exp Med 199:347-356.
7. Rappsilber J, Mann M, Ishihama Y (2007) Protocol for micro-purification, enrichment, pre-fractionation and storage of peptides for proteomics using StageTips. Nat Protoc 2:1896-1906.
8. Rappsilber J, Mann M, Ishihama Y (2007) Protocol for micro-purification, enrichment, pre-fractionation and storage of peptides for proteomics using StageTips. Nat Protoc 2:1896-1906.
9. Tyanova S, Temu T, Sinitcyn P, Carlson A, Hein MY, Geiger T, Mann M, Cox J (2016) The Perseus computational platform for comprehensive analysis of (prote)omics data. Nat Methods 13:731-740.

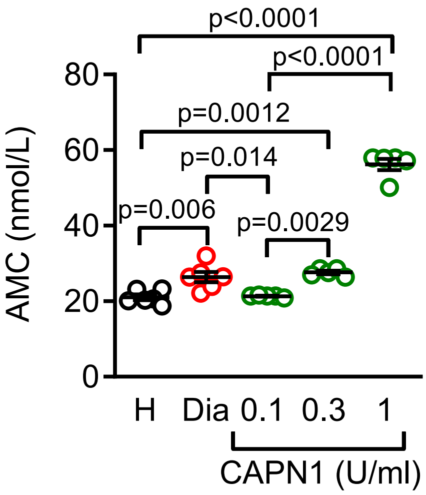

Online Figure 1. **Comparison of calpain activity in plasma and with recombinant CAPN1.** The activity of recombinant calpain as well as plasma from healthy and diabetic patients was measured by assessing the formation of the fluorescent metabolite 7-amino-4-methylcoumarin (AMC) from the calpain substrate N-succinyl-Leu-Leu-Val-Tyr-AMC. Calpain activity corresponds to the difference between OD measured with and without calpeptin and is expressed as AMC concentration; n=6 donors and 5 replicates of recombinant calpain. (one-way ANOVA and Tukey's post-test).

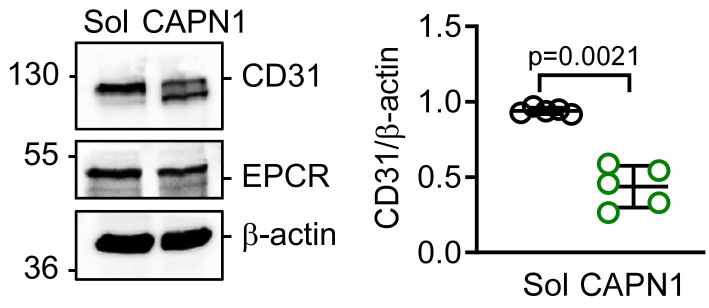

Online Figure 2. **Effect of calpain on EPCR and CD31 levels in endothelial cells.** Endothelial cell lysates were treated with solvent (Sol) or CAPN1 (0.3 U/ml, 30 minutes) and levels of CD31 and EPCR were assessed; n=5 different cell batches (Student's *t* test).

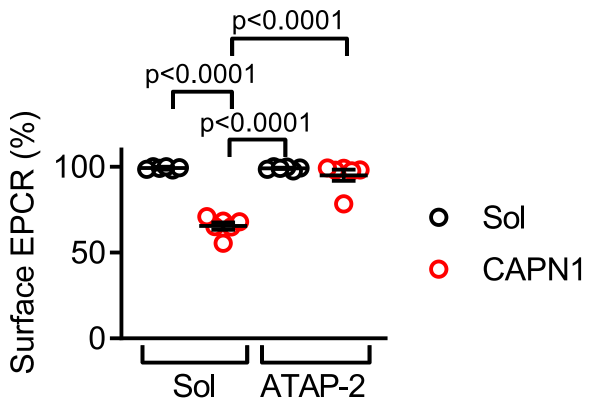

Online Figure 3. **Effect of PAR-1 neutralizing antibody** on **CAPN1-induced EPCR shedding.** EPCR levels on the surface of human endothelial cells treated with solvent (Sol) or CAPN1 (0.3 U/ml, 4 hours) in the absence or presence of ATAP-2; n=3 different cell batches in duplicate (two-way ANOVA and Tukey's post-test).

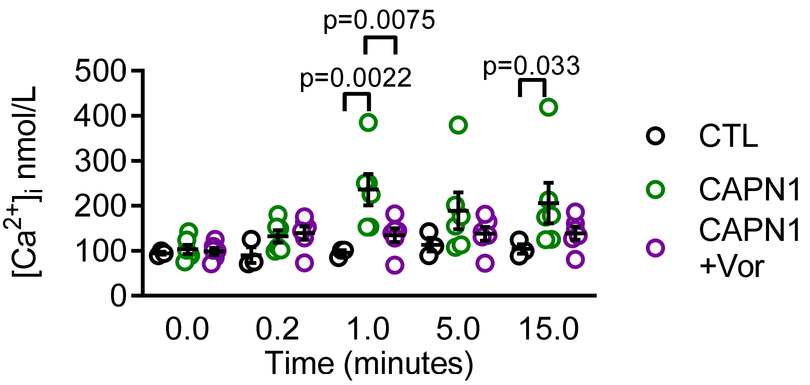

Online Figure 4. **Effect of extracellular CAPN1 on intracellular Ca^{2+} levels**. CAPN1 (0.3 U/ml) was added to fura-2-loaded human endothelial cells and changes in $[Ca^{2+}]_i$ assessed. Cells were either unstimulated or stimulated with CAPN1 in the presence of either solvent or the PAR-1 antagonist vorapaxar (Vor, 1 µmol/l); n= 5 different cell batches (2-way ANOVA and Tukey post-test).

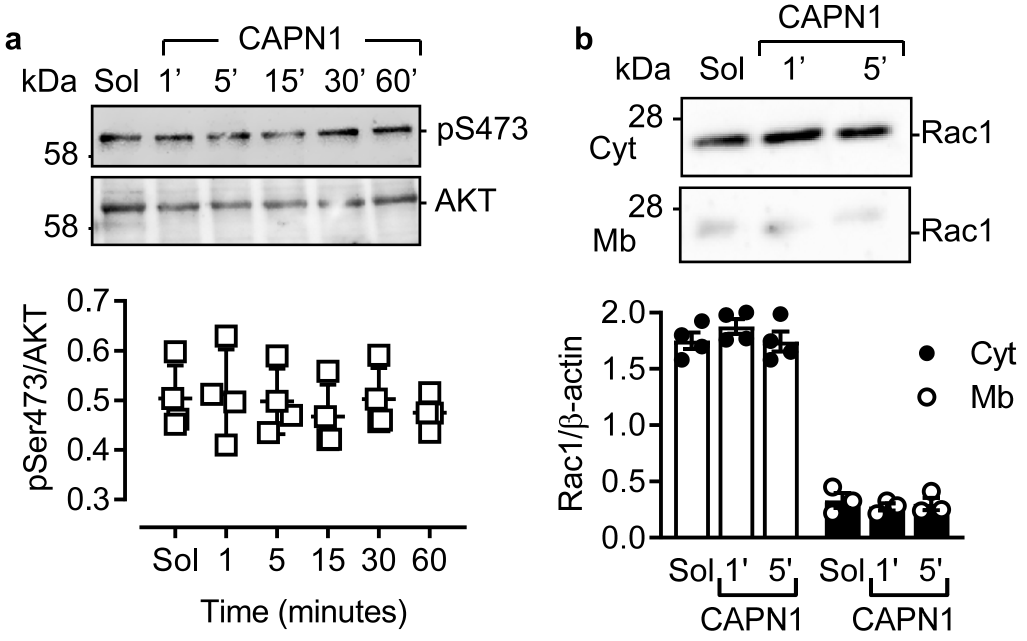

Online Figure 5. **Effect of extracellular CAPN1 on Akt and Rac1.** (**a**) Time-course of the effects of stimulation of human endothelial cells with CAPN1 (0.3 U/ml) on the phosphorylation of AKT on Ser473; n=4 different cell batches (one-way ANOVA and Tukey’s post-test); n.s compared to sol. (**b**) Levels of Rac1 in the cytosol (Cyt) and membrane fraction (Mb) generated from confluent cultures of endothelial cells treated with solvent or CAPN1 (0.3 U/ml) for up to 5 minutes; n=5 different cell batches (two-way ANOVA and Tukey’s post-test); n.s compared to sol within each group.

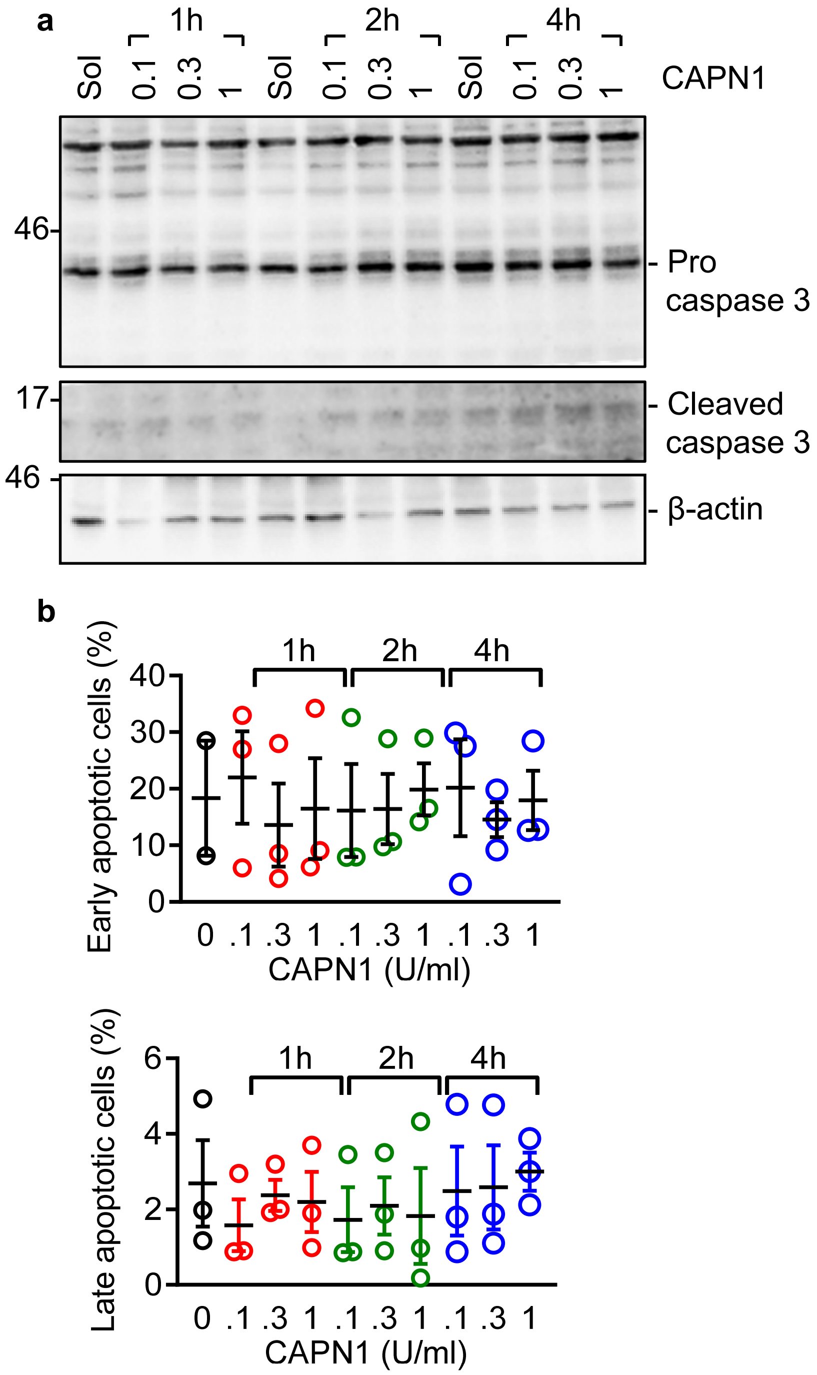

Online Figure 6. **Effect of extracellular CAPN1 on endothelial cell apoptosis**. Confluent cultures of human endothelial cells were treated with CAPN1 (0.1 to 1 U/ml for 1 to 4 hours). (**a**) Levels of full-length and cleaved caspase 3. (**b**) FACS analysis of early (FITC annexin V positive and propidium iodide negative) as well as late (FITC annexin V and propidium iodide positive) apoptosis markers; n=3 different cell batches (one-way ANOVA and Tukey's post-test); n.s while comparing the mean of each group to the mean of every other group.

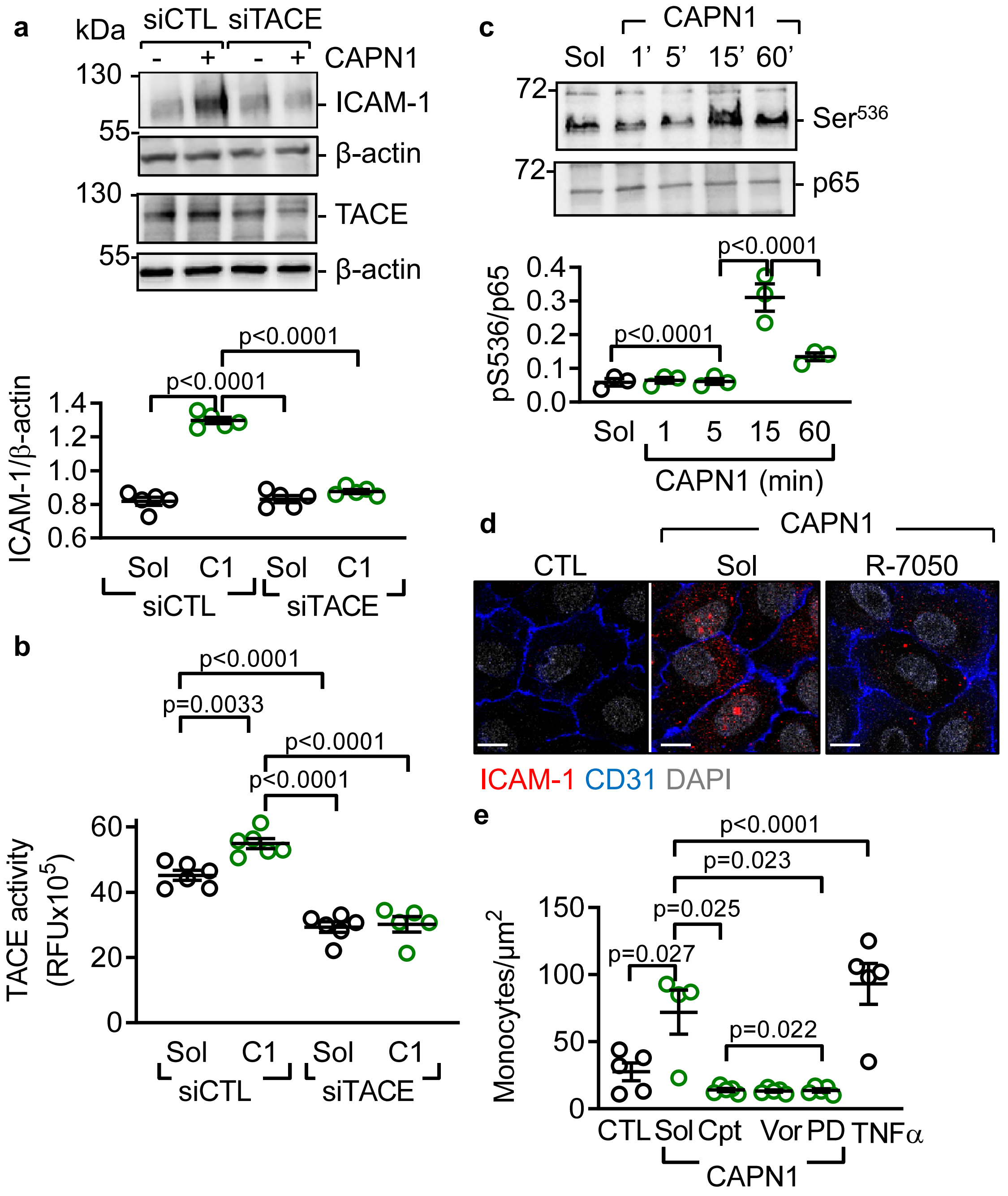

Online Figure 7. Effect of extracellular CAPN1 on endothelial cell activation. (**a**) Effect of CAPN1 (0.3U/ml, 4 hours) on the expression of ICAM-1 in human endothelial cells transfected with either scrambled siRNA (siCTL) or siRNA targeting TACE (siTACE). n=5 different cell batches. (**b**) Effect of CAPN1 (0.3 U/ml, 15 minutes) on TACE activity in human endothelial cells treated with either scrambled siRNA (siCTL) or siRNA targeting TACE (siTACE), n=6 different cell batches. (**c**) Time course of the effect of CAPN1 (0.3 U/ml) on the phosphorylation of p65 in human endothelial cells; n=3 different cell batches. (**d**) Effect of the TNF-α receptor antagonist (R-7050, 10 µmol/L) on the CAPN1 (0.3 U/ml, 4 hours)-induced surface expression of ICAM-1 (red) in human endothelial cells. CD31 = blue, DAPI =grey, bar = 10 µm. Comparable results were obtained using 4 additional cell batches. (**e**) Monocyte adhesion to mouse lung endothelial cells under basal conditions and after treatment with CAPN1 (0.3 U/ml, 4 hours) in the absence or in the presence of calpeptin (Cpt, 10 µmol/L), vorapaxar (Vor, 1 µmol/L) and PD98059 (PD, 10 µmol/L); bar=10 µm; n=5 different cell batches (one-way ANOVA and Tukey's post-test).

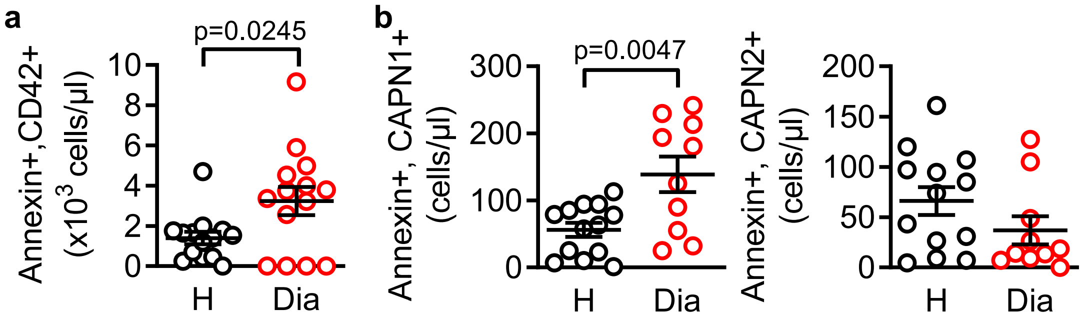

Online Figure 8. PMP and CAPN1 levels in plasma from nondiabetic and diabetic individuals. (**a**) FACS analysis of plasma from healthy donors and diabetic patients showing levels of platelet-derived microparticles; n=14 individuals pro group (Student's *t* test). (**b**) FACS analysis of the levels of active CAPN1 and CAPN2 on microparticles isolated from plasma of healthy donors and diabetic patients; n=13 healthy donors and 10 diabetic patients (Student's *t* test).

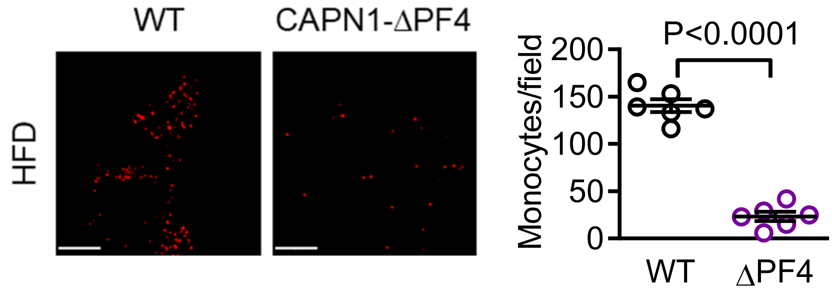

Online Figure 9. Reduced monocyte adhesion to aortae of CAPN1-ΔPF4 diabetic mice. Wild-type (WT) and CAPN1- ΔPF4 mice were made diabetic with a high-fat diet (HFD) over 20 weeks. Adhesion of freshly isolated and fluorescently labelled (red) murine monocytes from C57BL mice to isolated aortae; bar = 10 μm; n=6 animals per group (one-way ANOVA and Tukey's post-test).

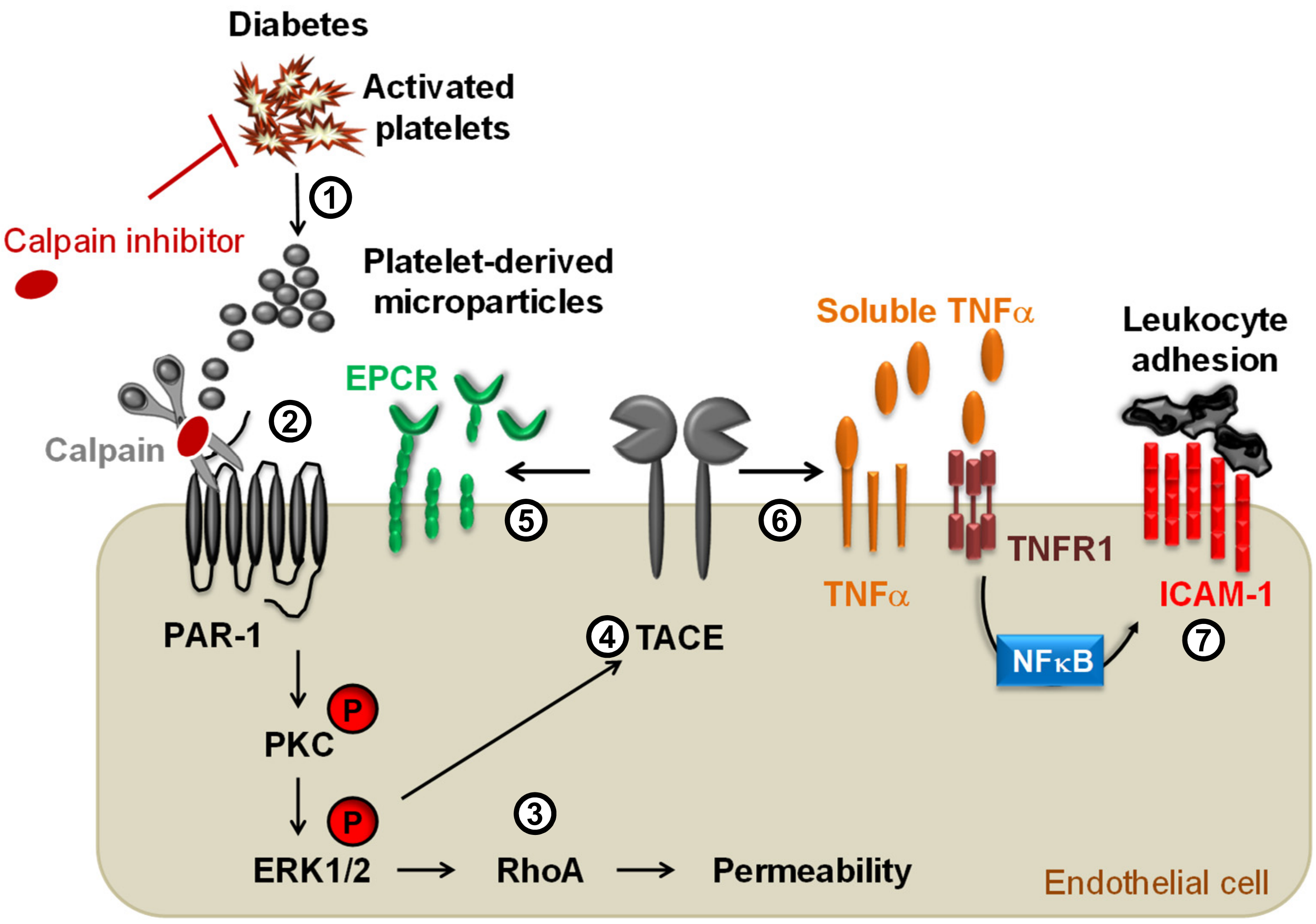

Online Figure 10. Proposed molecular mechanism of the vascular inflammation induced by platelet-derived CAPN1. (**1**) Platelets from diabetic patients are hyperactive and generate calpain-bearing microparticles. (**2**) Calpain cleaves the PAR-1 on the endothelial cells to activate intracellular signalling i.e. phosphorylation of PKC and ERK1/2. This is followed by (**3**) RhoA activation and enhanced cell permeability. At the same time, (**4**) the activation of the TNFα-converting enzyme (TACE) results in (**5**) EPCR shedding and (**6**) the generation of soluble TNF-α. The latter triggers (**7**) the expression of ICAM-1 in NFκB dependent manner and enhances monocyte adhesion to endothelial cells. Treatment with a calpain inhibitor prevents the calpain-induced vascular inflammation.
